# Monocyte Trafficking, Engraftment, and Delivery of Nanoparticles and an Exogenous Gene into the Acutely Inflamed Brain Tissue – Evaluations on Monocyte-Based Delivery System for the Central Nervous System

**DOI:** 10.1371/journal.pone.0154022

**Published:** 2016-04-26

**Authors:** Hsin-I Tong, Wen Kang, Philip M. C. Davy, Yingli Shi, Si Sun, Richard C. Allsopp, Yuanan Lu

**Affiliations:** 1 Office of Public Health Studies, University of Hawaii at Manoa, Honolulu, Hawaii, United Sates of America; 2 Department of Microbiology, University of Hawaii at Manoa, Honolulu, Hawaii, United States of America; 3 Institute for Biogenesis Research, University of Hawaii at Manoa, Honolulu, Hawaii, United States of America; Hungarian Academy of Sciences, HUNGARY

## Abstract

The ability of monocytes and monocyte-derived macrophages (MDM) to travel towards chemotactic gradient, traverse tissue barriers, and accumulate precisely at diseased sites makes them attractive candidates as drug carriers and therapeutic gene delivery vehicles targeting the brain, where treatments are often hampered by the blockade of the blood brain barrier (BBB). This study was designed to fully establish an optimized cell-based delivery system using monocytes and MDM, by evaluating their homing efficiency, engraftment potential, as well as carriage and delivery ability to transport nano-scaled particles and exogenous genes into the brain, following the non-invasive intravenous (IV) cell adoptive transfer in an acute neuroinflammation mouse model induced by intracranial injection of *Escherichia coli* lipopolysaccharides. We demonstrated that freshly isolated monocytes had superior inflamed-brain homing ability over MDM cultured in the presence of macrophage colony stimulating factor. In addition, brain trafficking of IV infused monocytes was positively correlated with the number of adoptive transferred cells, and could be further enhanced by transient disruption of the BBB with IV administration of Mannitol, Bradykinin or Serotonin right before cell infusion. A small portion of transmigrated cells was detected to differentiate into IBA-1 positive cells with microglia morphology in the brain. Finally, with the use of superparamagnetic iron oxide nanoparticles SHP30, the ability of nanoscale agent-carriage monocytes to enter the inflamed brain region was validated. In addition, lentiviral vector DHIV-101 was used to introduce green fluorescent protein (GFP) gene into monocytes, and the exogenous GFP gene was detected in the brain at 48 hours following IV infusion of the transduced monocytes. All together, our study has set up the optimized conditions for the more-in-depth tests and development of monocyte-mediated delivery, and our data supported the notion to use monocytes as a non-invasive cell-based delivery system for the brain.

## Introduction

Monocytes and monocyte-derived macrophages (MDM) possess broad homeostatic, immune sensing and surveillance functions [1, 2]. Their ability to traffic through circulation and accumulate precisely at the diseased sites makes them an attractive tool for drug carriage and gene delivery [3–7]. The need for cell-based delivery systems is immediate in order to combat central nervous system (CNS) diseases, because many therapeutic compounds and biologics are known to have limited capability to penetrate the blood-brain barrier (BBB) or to reach sites further from their administration points effectively [8].

Early studies using hematopoietic stem cell (HSC) transplanted into lethally irradiated animals demonstrated that blood circulating monocytes were recruited to the CNS and differentiated into resident macrophages and microglia cells once reaching their destinations [9–11]; whereas recent studies have suggested the use of lethal irradiation induced additional damages to the CNS, hence overestimated the true ability of monocyte to infiltrate and differentiate into resident microglia cells [12, 13]. Nevertheless, recruitments of circulating monocytes to the diseased sites within the CNS were evident in numerous neurological disorders [6, 14–17]. Therefore, the use of monocytes and MDM for precise therapeutics delivery still holds great promises for combating many CNS disorders, including Parkinson’s and Alzheimer’s Diseases, Multiple Sclerosis, and HIV-associated neurocognitive disorders [3, 5–7].

Comparing to bone marrow transplants (BMT) using precursor cells, adoptive transfer of differentiated cells avoids the involvement of lethal irradiation, and is a relatively risk free procedure with minimum side effects [18–20]. Thus, by exploring the migration property of IV transferred monocytes and MDM to regions of interests, it is possible to selectively transport disease combating genes or medicines to inflamed or damaged sites in the brain in a non-invasive fashion. Thus far, a number of studies have been conducted to test monocytes- and MDM-mediated delivery of nano-formulated medicines and therapeutic genes into the CNS [3, 5–7], but the optimum conditions for such delivery system has not been fully established. In order to carry out effective treatment functions, therapeutics-carriage cells need to be present at target sites in high numbers. Therefore, identifying suitable cellular sources to be used as transporting vehicles, and developing methods to increase cell vehicle target site concentration are essential for establishment of a cell-based delivery system [21]. Both freshly isolated monocytes and culture-expanded MDM (cMDM) were tested for their capability to reach the CNS following adoptive transfer [3, 5–7, 22], but no quantitative comparison have been performed to evaluate the suitable cellular source for transporting therapeutic agents to the brain. Hence, this study was aimed to establish the optimized conditions for the non-invasive cell-based delivery system, including testing and determining the homing efficiency of freshly isolated monocytes and cMDM to the inflamed brain regions, establishing conditions that could enhance the cell vehicle concentration at the target sites, assessing the potential of these recruited cells to engraft and differentiate in the brain, and validating the ability of the cell vehicles to carry”cargos” into the brain following systemic IV adoptive transfer.

Using a mouse model of acute brain subregional inflammation induced by intracranial (IC) injection of *Escherichia coli (E*. *coli)* Lipopolysaccharide (LPS), we have demonstrated that freshly isolated monocytes have high inflamed-brain homing efficiency following IV adoptive transfer, and have established the conditions for enhanced cell transmigration and increased cell vehicle concentrations in brain target sites by increasing IV infusion cell amount and transiently disrupting the BBB using chemical agents including Mannitol, Bradykinin, and Serotonin. In addition, we have observed that a small portion of the recruited monocytes were able to differentiate into IBA-1 positive cells with microglia morphology in the brain. Furthermore, with the use of superparamagnetic iron oxide nanoparticle (SPION) SHP30, the ability of nanoscaled agent-carriage monocytes to enter the inflamed brain region was validated. In addition, lentiviral vector (LV) DHIV-101 (D101) was utilized to mediate the transfer of green fluorescent protein (GFP) gene into monocytes in this study, and the presence of GFP gene was detected in the brain of affected animals 2 days after IV infusion of the LV-transduced monocytes.

All together, our study has set up the optimized conditions for the more-in-depth study of moncoyte-mediated delivery system, and our new data supports the notion of using monocyte migration as a means of therapeutics delivery into the brain—with the ultimate goal of affecting neuronal disease outcomes.

## Materials and Methods

### Animals

Six- to 10-week old male and female C57BL/6 mice (Obtained from Animal and veterinarian services, University of Hawaii at Manoa) and male green fluorescent protein (GFP) transgenic male C57BL/6-Tg (UBC-GFP) 30Scha/J mice (The Jackson Laboratory, stock #004353) were acquired for this study. C57BL/6 mice were used in all experiments except for immunohistochemical (IHC) and immunofluorescent studies, which GFP transgenic mice were used as cell donors and C57BL/6 mice as recipients. In all studies, male mice were used as cell donors, and female mice as recipients. All mice were bred and maintained in the animal facility at University of Hawaii at Manoa campus by following the institutional guidelines for the humane care and investigation of laboratory animals, and all animal studies were reviewed and approved by the Institutional Animal Care and Use Committee (IACUC) of University of Hawaii at Manoa (Protocol # 09–767). In all experiments, mice were monitored twice daily throughout the experimental periods. A humane endpoints protocol was included in this study, and experimental animals were to be euthanized if they displayed inability to reach food or water for more than 24 hours, and if more than 20% decrease in body weight was detected. No early death was observed in any animal prior to the experimental endpoints in this study.

### Isolation and Cultivation of Mouse Monocytes and cMDM

Eight- to 10-week old male mice were used as cell donors. Mouse cMDM were prepared as described previously [5]. In brief, bone marrow cells (BMC) were isolated from donor mice femur and tibia of the hind limbs. Erythrocytes were removed by lysis with ACK lysis buffer (Quality Biological, Inc.), and a single cell suspension was obtained by passing through 40-μm cell strainers (BD Falcon, #352340), and was either resuspended in PBS and used for cell IV infusion, or cultured in suspension with complete growth medium composed of RPMI1640 medium supplemented with 10% FBS and 1,000 U/ml M-CSF (obtained from 5/9 m alpha3-18 cell media, ATCC#CRL-10154. M-CSF in condition media was quantified using Human M-CSF quantikine ELISA kit, R&D system cat#DMC00B), at 37°C with 5% CO_2_ for 2, 5, 7, 9, 12, 15, 21 and 28 days prior to flow cytometry analysis or adoptive transfer study.

In studies using freshly isolated monocytes (Enriched monocytes, EnMo), monocytes were enriched from the freshly isolated BMC suspension using EasySep mouse monocyte enrichment kit (Stem Cell Technologies, #19761) according to manufacturer’s instructions.

### Flow Cytometry

*In vitro* monocyte to macrophage differentiation was assessed in cMDM at 0, 2, 5, 7, 9, 12, 15 and 21 days post isolation by fluorescence-activated cell sorting (FACS) with a FACSAria III, and cytometry data were analyzed using FACSDiva v. 6.1 (BD Biosciences). Cells were collected from suspension culture flask, washed with PBS, and incubated with antibodies (details below) for 30 minutes on ice. Unbound antibodies were removed by washing cells with PBS. Recovered cell pellets were resuspended in 1% PFA in PBS and stored at 4°C until analysis. Cell samples were stained with the following dye and antibodies for FACS analysis according to the manufacturers recommendations: Zombie Green (1:500, BioLegend cat#423111) for viability assessment, rat anti-CD11b (1:500, PE-Dazzle594 conjugated, BioLegend cat#101255), rat anti-Ly6c (1:200, PerCP conjugated, BioLegend cat#128027), monoclonal rat anti-CCR2 (1:20, APC conjugated, R&D Systems cat#FAB5538A), and rat anti-F4/80 (1:40, APC-Cy7 conjugated, BioLegend cat#123117).

Freshly isolated BMC were used in single stain controls to create spectral compensation, and in florescence minus one (FMO) controls to establish appropriate gating strategies. Forward and side scatter properties were applied in gating to exclude doublets and cell debris, and the single cell population was assessed for their viability in the FITC channel (Zombie Green viability assessment). CD11b+ cells (Zombie Green^neg^) were then divided into three populations based on combined assessment of their expression of F4/80 and Ly6c. The F4/80^high^Ly6C^neg^, F4/80^low^Ly6C^low^, and F4/80^med^Ly6c^high^ populations in this plot were labeled as macrophages, Ly6C^low^ monocytes, and Ly6C^high^ monocytes, respectively. CCR2 expression was assessed for each of these three populations separately, Ly6C^high^ monocytes were positive, Ly6C^low^ monocytes and macrophages were low to negative. The relative percentages of each cell population within the samples were generated from statistical analysis of the F4/80 –Ly6c plots.

EnMO was further analyzed for their expression of CD115 (rat anti-CD115, APC conjugate, 1:100, BioLegend #135509), CD11b+, F4/80, and Ly6C to confirm the purity of the enriched cells.

### *E*. *coli* LPS-Induced Acute Brain Inflammation and Cell IV Adoptive Transfer

Six- to 8-week old female mice were anesthetized with Avertin (2,2,2-Tribromoethanol, 250 mg/kg, IP) and positioned on a stereotaxic apparatus (Stoelting co.). *E*. *coli* LPS (Serotype O111:B4, S-form. Enzo Life Sciences, ALX-581-M005) was administered into the right hemisphere (AP 0.0 mm, ML +2.5 mm, DV -4.0 mm from bregma) to induce acute subregional neuroinflammation in the brain. Each animal received 5 μg of *E*. *coli* LPS in 5 μL solution or 5 μL of PBS (Sham) over a period of 5 minutes. PBS-injected animals and the un-injected contralateral hemisphere were used as controls. At 24 hrs following ICI delivery of LPS, 100–150 μL cell suspension containing cells (5x10^6^ if not otherwise indicated) or PBS (Sham) were injected into the lateral tail vein of recipient mice. Mice were sacrificed at day 1, 2, 3, 5, 7 or 14 after cell infusion for analysis.

### LV-Mediated GFP Gene Transfer into Monocytes

The HIV-1-based defective LV system D101 was used for transduction of monocytes [23–26]. The vector was produced by transient transfection in HEK293T cells by calcium phosphate precipitation method with a packaging construct pCMV-ΔR8.2, an envelop construct pCMV-VSV-g, and a transfer construct pD101 which contained the reporter gene GFP, as described previously [24]. Transduction of monocytes was done by spin-infection. 2 × 10^7^ transducing units (as titrated on HEK293T cells) D101 vectors were incubated with polybrene (final concentration 8 μg/mL) at RT for 10 minutes before combined with 1 × 10^6^ freshly isolated, enriched monocytes (MOI = 20) in serum free medium. The cell/vector/polybrene mix is then transferred to a 5mL round-bottom polystyrene tube, and transduction was carried out by centrifuging the culture at 1,500 ×g for 90 minutes at 32°C. Cells were then washed three times by centrifugation with PBS to remove excess vector and polybrene, and immediately injected IV into recipient animals. A small portion of the transduced cells were maintained *in vitro* for 7 additional days following in complete culture medium, and the efficiency of monocyte transduction was determined by counting the number of GFP-positive cells at random fluorescent microscope fields.

### Monocyte Uptake of SHP30

Commercially available SPIO nanoparticle (NP) SHP-30 (Ocean NanoTech) was used in this study. SHP30 is 30 nm in core diameter (38–40 in hydrodynamic diameter), has oleic acid and amphipilic polymer coating with carboxylic acid reactive group, and with a Zeta potential range from -30mV to -50mV. Immediately following isolation and enrichment, monocytes were transferred to 5mL round-bottom PS tubes, and cultured in suspension with complete culture medium containing 50 μg/mL SHP30, for 12 to 14 hours. The cells were then washed 3 times with PBS by centrifugation to remove extracellular SHP30, and injected IV into recipient animals. The SHP30 uptake efficiency was 100% as determined by Prussian blue staining.

### Trypan Blue Exclusion Assay

Following LV transduction or SHP30 uptake, the cell viability of control and carrier monocytes was measured with Trypan blue exclusion assay, by mixing equal volume of cells and 0.4% trypan blue solution (Sigma, Cat#T8154). Cells stained blue were considered dead, and unstained cells were considered viable. The final cell viability was presented in percentage, as comparing to the viability of control monocytes (no gene or SHP30 carriage) set as 100%.

### Real-Time qPCR

Genomic DNA was isolated from brain regions ± 1.0 mm from the LPS injection site (average tissues weight ranged from 50–60 mg) using QIAamp DNA mini kit (Qiagen) following the manufacturer’s instructions. The amount of donor-derived cell DNA was quantitatively determined by real-time qPCR with primers and probe specific to male murine Y chromosome [27]. Real-time qPCR was performed on an iQ5 optical system (BioRad) using forward primer 5’-TTTTGCCTCCCATAGTAGTATTTCCT-3’, reverse primer 3’-TGTACCGCTCTGCCAACCA-3’ and the TaqMan probe 5’-/56-FAM/AGGGATGCC/ZEN/CACCTCGCCAGA-/3IABkFQ/-3’ (Integrated DNA Technologies). Standard curves were generated by serially diluting DNA from male mouse brain. DNA isolated from female mouse brain was included as a negative control in every assay. The average DNA content in a diploid mouse cell range from 5 to 7 pg [28], therefore a parameter of 6 pg gDNA per cell was applied to convert DNA to cell numbers presented in all results.

### PCR

The presence of GFP gene delivered by D101 transduced monocytes to the brain were detected with conventional PCR method using forward primer F-GFPreal: 5’-GGTGAGCAAGGGCGAGGAG-3’, and reverse primer R-GFPreal: 5’-GCCGGTGGTGCAGATGAACT-3’. PCR was performed with a MasterCycler Gradient (Eppendorf, Germany). Five microliter of inflamed brain tissue DNA was combined with 20 μL of a mixture containing 1X Taq (Mg^2+^ free) reaction buffer (New England Biolabs, NEB, MA), 1.5 mM MgCl_2_ solution (NEB, MA), 200 nM of each dNTPs (Sigma-Aldrich, MO), 400 nM of each primer (Integrated DNA technologies, IA), and 2 units of Taq polymerase (provided by Dr. Huang, University of Hawaii at Manoa). The Amplification started with an initial denaturation at 94°C for 5 min, followed by 30 cycles of denaturation at 94°C for 30 sec, annealing at 56°C for 30 sec, extension at 72°C for 30 sec, and a final extension at 72°C for 5 min. PCR products were subjected to 2% agarose gel electrophoresis, alongside a 50 bp DNA marker (NEB, MA), stained with ethidium bromide (EtBr) and viewed with the Molecular Imager Gel Doc XR+ system (BioRad Laboratories, Inc., CA).

### Analysis of Cytokine Transcripts by Real-Time RT-PCR

Tissue RNA was isolated from the inflamed and control brain region by TRIZOL reagent (Life technology) at 72 hrs post ICI of LPS or PBS (48 hrs post cell IVI). Reverse transcription (RT) was performed with iScript Reverse Transcription Supermix for RT-qPCR (Bio-Rad laboratories), followed by quantitative real-time PCR with specific primer sets (Table 1) using iQ SYBR Green Supermix (Bio-Rad laboratories) through an iQ5 optical system (Bio-Rad). Results were analyzed for relative gene expression using the 2-delta-delta CT method.

**Table 1:** Real-time RT-PCR primer sequences for cytokine transcription profile.

Gene name	GeneBank Accession #	Oligonucleotide sequences	Reference
GAPDH	NM_008084	forward: 5’–CTCCACTCACGGCAAATTCAA–3’ reverse: 5’–GATGACAAGCTTCCCATTCTCG–3’	[7]
TNFα	NM_013693	forward: 5’–CCGTCAGCCGATTTGCTATCT–3’ reverse: 5’–ACGGCAGAGAGGAGGTTGACTT–3’	[7]
IL-1β	NM_008361	forward: 5’–ACAACAAAAAAGCCTCGTGCTG–3’ reverse: 5’–CCATTGAGGTGGAGAGCTTTCA–3’	[7]
IFNγ	NM_008337	forward: 5’-ACAGGTCCAGCGCCAAGCAT-3’ reverse: 5’-ACCCCGAATCAGCAGCGACT -3’	This study
TGFβ1	NM_011577	forward: 5’–AGGACCTGGGTTGGAAGTGG–3’ reverse: 5’–AGTTGGCATGGTAGCCCTTG–3’	[7]
IL-10	NM_010548	forward: 5’–AGGCGCTGTCATCGATTTCTC–3’ reverse: 5’–TGCTCCACTGCCTTGCTCTTA–3’	[7]
NOS2	NM_010927	forward: 5’–GGCAAACCCAAGGTCTACGTTC–3’ reverse: 5’–TACCTCATTGGCCAGCTGCTT–3’	[7]
IL-2	NM_008366	forward: 5’-GCATGCAGCTCGCATCCTGT-3’ reverse: 5’-TGCTGCTGTGCTTCCGCTGT-3’	This study
IL-4	NM_021283	forward: 5’–CACGGATGCGACAAAAATCA–3’ reverse: 5’–CTCGTTCAAAATGCCGATGA–3’	[7]
IL-12p35	NM_008351	forward: 5’–AAATGAAGCTCTGCATCCTGC–3’ reverse: 5’–TCACCCTGTTGATGGTCACG–3’	[7]
IL-12p40	NM_008352	forward: 5’-ACCAGGCAGCTCGCAGCAAA-3’ reverse: 5’-ACACATCCCACTCCCACGCT-3’	This study
BDNF	NM_007540	forward: 5’–AGGCACTGGAACTCGCAATG–3’ reverse: 5’–AAGGGCCCGAACATACGATT–3’	[7]
GDNF	NM_010275	forward: 5’–GGGTGCGTTTTAACTGCCAT–3’ reverse: 5’–GCCCAAACCCAAGTCAGTGA–3’	[7]

### Transient Disruption of the BBB by Chemical Agents

Twenty-four hours after LPS injection (5 μg, IC), female recipient mice were anesthetized with Avertin (125 mg/kg, IP), and different transient BBB disrupting (BBBD) agents were administered prior to IVI of 5×10^6^ monocytes (see below). Dosages and injection time points of the BBB disrupting agents were selected from previously published methods, with some modifications [22, 29–32]. Detailed descriptions for each BBB disrupting agent usage are as follows: 1) D-Mannitol (Sigma, cat#M9546): 200 μL of 25% (w/v), IV, with IV delivery of donor cells 8 minutes later; 2) L(+)Arabinose (Calbiochem, cat#78680): 200 μL of 1.8M, IV, with IV delivery of donor cells immediately thereafter; 3) Bradykinin (Sigma #B3259): 200μl of 1g/L, IV, with IV delivery of donor cells 20 minutes later; 4) 5-Hydroxytryptamine creatinine sulfate monohydrate (Serotonin creatinine sulfate) (MP Biomedicals, 151315): 200 μL of 1g/L, IV, with IV delivery of donor cells immediately thereafter. 5) Cyclosporin A (Enzo Life Sciences, 380–002): 50 mg/kg, Subcutaneous (SQ), injected right after LPS ICI, followed by IV delivery of donor 24 hrs later. For the BBB disrupting agents administered by IV injection, monocytes were infused into the alternative tail vein at indicated time points as described above. Animals that received cells only (with no BBB disrupting reagents) at 24 hrs post ICI delivery of LPS were used as controls (value set at 100%), and the relative amount of recruited donor cells from the test groups was quantified and comparatively analyzed with the control groups using unpaired student t-test, and one-way ANOVA among all test groups.

### IHC and immunofluorescent Analyses

Twenty-four hours post ICI of 5 μL LPS (1 μg/μL) or sham (PBS), female C57BL/6 recipient mice received IVI of 5×10^6^ GFP-positive enriched monocytes from male donor C57BL/6-tg (UBC-GFP) 30Scha/J mice. At days 2, 5, 7 and 14 post cell infusion, recipient mice were sacrificed by intracardiac perfusion with PBS followed by 4% paraformaldehyde (PFA) in PBS. Brain tissues were collected and post-fixed in 4% PFA overnight at 4°C prior to dehydration in gradient sucrose solutions. Brain tissues were then embedded in OCT compound and frozen in 2-methylbutane cooled on dry ice. Serial coronal brain sections of 25-μm were prepared from sample tissue ±1.0 mm of the injection point. Donor-derived GFP-positive cells were detected with goat anti-GFP antibody (1:200, Rockland #600-101-215). Polyclonal antibody to ionized calcium-binding adaptor molecule 1 (Iba-1, 1:400, Wako Pure Chemical Industries, Ltd. Japan. Stock#01919741) was used to identify mice brain microglial cells. Astrocytes were detected with Rabbit polyclonal Abs against glial fibrillary acidic protein (GFAP, 1:1,000, DaKo Cytomation #Z0334). Secondary antibody conjugated with Rhodamine was used for fluorescent imaging (Goat anti-Rabbit IgG with Rhodamine conjugate, 1:200, Jackson Immuno Research; Donkey anti-Goat IgG with Rhodamine conjugate, 1:200, Rockland #605-700-002). For IHC detection of GFP expressing cells, Donkey anti-Goat IgG with Biotin conjugate was used (1:200, Rockland). In addition to IHC, the presence of SHP30 within the cytoplasm of recruited donor cells in the brain was detected by Prussian blue staining, and counter stained with Nuclear Fast Red solution (Sigma). All microscope images were viewed with a Nikon eclipse TE2000-U epi-fluorescence microscope (with 4x, 10x, and 20x objective lenses, total magnifications of 40x, 100x, and 200x, respectively), which was equipped with a CoolSNAP ES2 CCD camera (for fluorescent image acquisition), and with a QIClick^™^ CCD Camera (for bright-field color image acquisition). All microscope image were captured at room temperature (25°C) using NIS-elements BR2.30 software, images were further processed with Adobe Photoshop CS3 (version 10.0.1) and Image J (1.48V).

### Statistic Analysis

For all experiments, final data presented were obtained from 3 to 6 animal for each test group, and were represented as mean values ± SD. Analysis of variance (ANOVA) was used to analyze studies with three or more experimental groups. Unpaired t test was used to analyze studies between two experimental groups. Pearson correlation coefficient was used to calculate correlation (R) and coefficient (R^2^). Results with *p-value* < 0.05 were considered significant.

## Results

### Migration Efficiency of IV Adoptive Transferred Monocytes and cMDM to Acutely Inflamed Brain Tissues

In all tests, male mice were used as cell donors, and female mice as recipients. This sex-mismatched system allowed tracking and quantifying of recruited donor cells in the brain by detecting the amount of y-chromosome DNA [27]. Our initial experiments demonstrated that the amount of accumulated donor cells in the inflamed brain region was the highest when cells were introduced to recipient animals at 24 hrs following LPS ICI, and when brain tissues were collected at 48 hrs post cell IV infusion (S1 Fig). These test conditions were used in all experiments of this study unless otherwise indicated.

**Fig 1:**
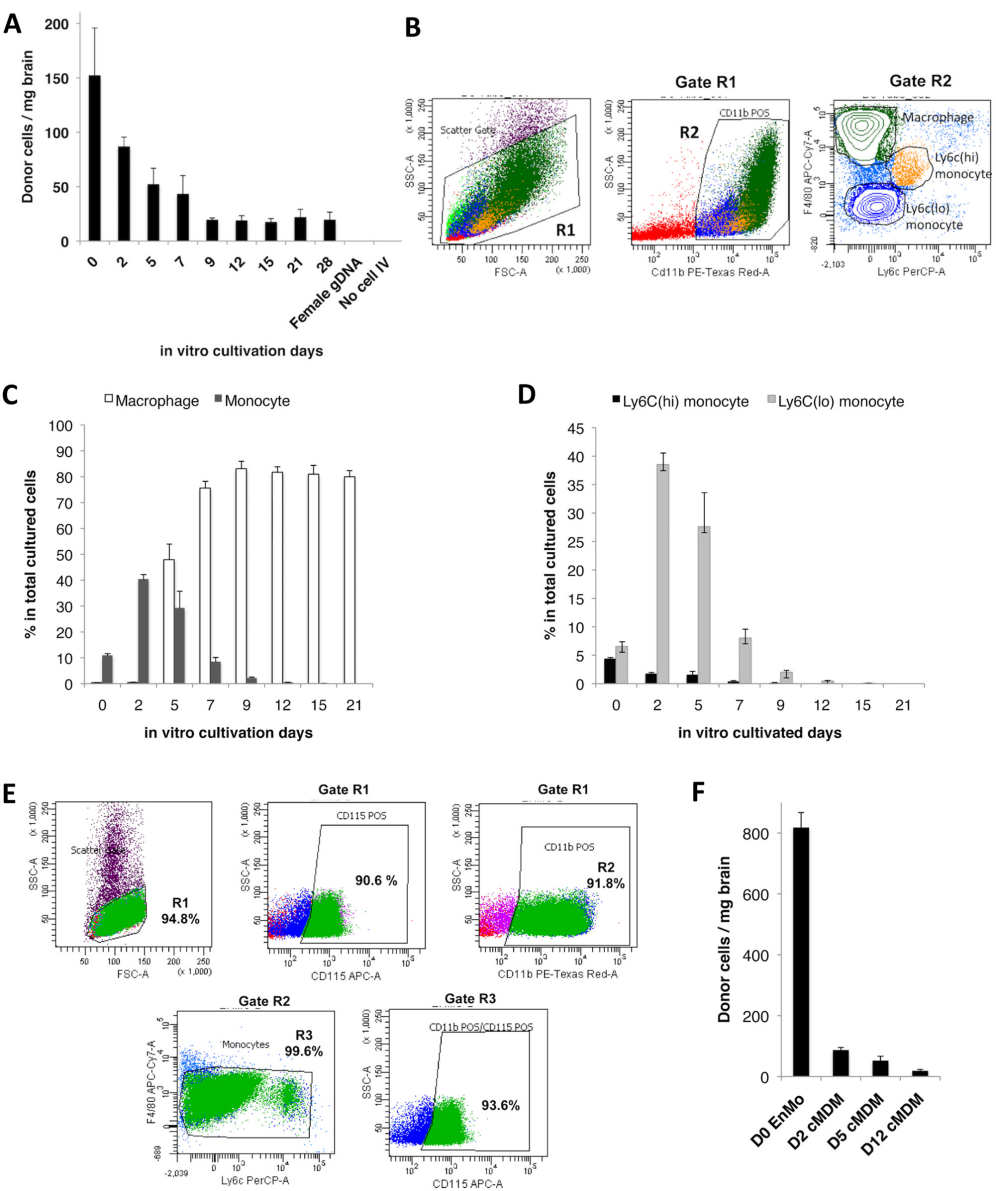
Migration efficiency of IV transferred monocytes and cMDM to acutely inflamed brain tissue is linked to monocyte-to-macrophage differentiation. (A) MDM cultured from day 0 to day 28 were introduced IV (5×10^6^ cells) into recipient mice bearing LPS-induced acute neuroinflammaiton, and cMDM *in vivo* brain homing efficiency deceased as the *in vitro* cultivation time went up. Female gDNA = female mouse brain tissue genomic DNA (negative control). No cell IV = LPS injected control animal that received no cell IV transplant. Final data presented here represents mean values ± SD. Data was analyzed by one-way ANOVA, with resulting p-value <0.05. (B–D) Flow cytometry analysis on cMDM cell populations at different time points post *in vitro* cultivation. (B) Example of phenotype identification of cMDM using D5 cMDM. The green cluster reflects macrophage phenotype (CD11b^+^ F4/80^hi^ Ly6C^low to neg^), blue cluster reflects Ly6c^low^ monocytes (CD11b^+^ F4/80^low to med^ Ly6C^low to neg^), and yellow cluster reflects Ly6C^hi^ monocytes (CD11b^+^ F4/80^low to med^ Ly6C^high^). (C) The ratio of macrophages (CD11b^+^ F4/80^high^ Ly6C^neg^) and total monocytes (CD11b^+^ F4/80^low to med^ Ly6C^low to high^), and (C) the ratio of Ly6C^hi^ monocytes (CD11b^+^ F4/80^med^ Ly6C^high^ CCR2^+^) and Ly6C^lo^ monocytes (CD11b^+^ F4/80^low^ Ly6C^low^ CCR2^low to neg^) in culture were evaluated. (E) Phenotyping of freshly isolated EnMO by flow cytometry analysis. Expression level of CD115, CD11b, F4/80, and Ly6c were measured to determine the purity of the enriched cells. (F) Freshly isolated EnMO showed superior brain homing efficiency over cMDM. 5x10^6^ of EnMO (D0 EnMO), or MDM cultured for 2-, 5-, or 12- days (D2 cMDM, D5 cMDM, D12 cMDM, respectively) were infused IV to animals with acute neuroinlfammation, and the number of donor cells present in the LPS-injected brain hemisphere was quantified at 48 hour following cell IVI. Final data presented here represents mean values ± SD. Data was analyzed by one-way ANOVA, with resulting p-value all < 0.05.

Both freshly isolated monocytes and cMDM have been tested in adoptive transfer studies to reach target tissues with various degree of success [3, 5–7, 22, 33, 34]. To determine the more suitable cellular sources as delivery vehicles for the brain, MDM were cultivated *in vitro* in the presence of M-CSF for 0 to 28 days prior to their IV infusion. As the results, freshly isolated cells (BMC) showed the highest migratory efficiency, even though only ~10% of the total cell population was monocytes in origin **(**Fig 1A and 1B) [35]. The cell transmigration efficiency decreased with the cultivation time—the efficiency dropped almost by half when 2-day’s cultures were used, and continued to drop until a plateau was reached with cells cultured for 9 days and thereafter (Fig 1A).

To explain the decreased brain homing efficiency *in vivo*, cMDM population was analyzed for cell types at corresponding cultivation time points. The frequency of macrophages (CD11b^+^ F4/80^high^ Ly6C^neg^ CCR2^neg^) and monocytes (including Ly6C^hi^ monocytes as CD11b^+^ F4/80^med^ Ly6C^high^ CCR2^+^, and Ly6C^lo^ monocytes as CD11b^+^ F4/80^low^ Ly6C^low^ CCR2^low to neg^) was measured with flow cytometry (Fig 1B). Freshly isolated BMC was composed of 10% monocytes and less than 0.4% macrophages (Fig 1C). During *in vitro* cultivation, macrophage numbers increased quickly at day 5, and maintained at a consistent 80% of the total cell population (including all cells present in culture flask), or at > 90% among the CD11b^+^ cell population, from day 9 and thereafter. Conversely, total number of monocytes only showed a brief increase that peaked at day 2, followed by rapid decrease and fell below 0.5% by day 12 and thereafter.

Nevertheless, even with the presence of a much higher percentage of total monocytes, *in vivo* transmigration of day 2- and day 5- cMDM into the brain was not as efficient as the freshly isolated BMC (d0) (Fig 1A). Further analysis revealed that majority of the monocyte population at these later time points was composed of Ly6C^lo^ monocytes (Ly6C^low^ CCR2^low to neg^); whereas the portion of Ly6C^hi^ monocytes (Ly6C^high^ CCR2^+^) decreased over time (Fig 1D). Previous studies have reported that Ly6C^high^, but not Ly6C^low^ monocyte, are actively recruited to inflammatory sites [34, 36, 37], and Ly6C^hi^ monocytes are known to appear in circulation in large quantities during both acute and chronic inflammation [38]. Hence, this subtype of the donor cells was likely to be the primary ones entering the acutely inflamed brain following IV administration. Thus, the rapid decline of this cell population during *in vitro* cultivation would explain why the efficiency of cell transmigration into the inflamed brain tissue declines with prolonged culture time.

A previous study has demonstrated that infiltrating neutrophiles quickly appeared in the inflamed brain regions but died within 18 hours following LPS IC inoculation, and monocytes were the major infiltrating CD11b+ cells detected in the brain after 24 hours [39]. Nevertheless, in this study, the recruited donor-derived cells in the brain might come from cell types other than monocytes and macrophages, since freshly isolated BMC contains mixed cell populations, and cMDM was only partially purified by *in vitro* cultivation with the use of M-CSF. To ensure the cell purity in subsequent studies, monocytes were further enriched from the freshly isolated BMC using a negative selection method. Results from flow analysis have confirmed that over 91% of EnMO (SSC^low^) were positive for CD11b, and over 90% were positive for the monocyte-macrophage lineage marker, CD115 (Fig 1E). In addition, over 99% of CD11b^+^ cells were found to be F4/80^med-low^, and over 93% of the CD11b^+^F4/80^med-low^ population were CD115^+^ (Fig 1D). Furthermore, animals received five million of EnMo showed a significant increase in numbers of transmigrated cells in the brain: a 9.4-fold, 15.6-fold and 43-fold increase was observed as compared to animals that received the same amount of d2-, d5- and d12- cMDM (Fig 1F). Confirmed with its superior inflamed-brain homing efficiency, freshly isolated monocyte was deemed to be the better candidate as cellular vehicles for the brain, and was used in all subsequent tests.

### IV Adoptive Transferred Monocyte Ingression in the Inflamed Brain Did Not Aggravate Neuroinflammation

**Fig 2:**
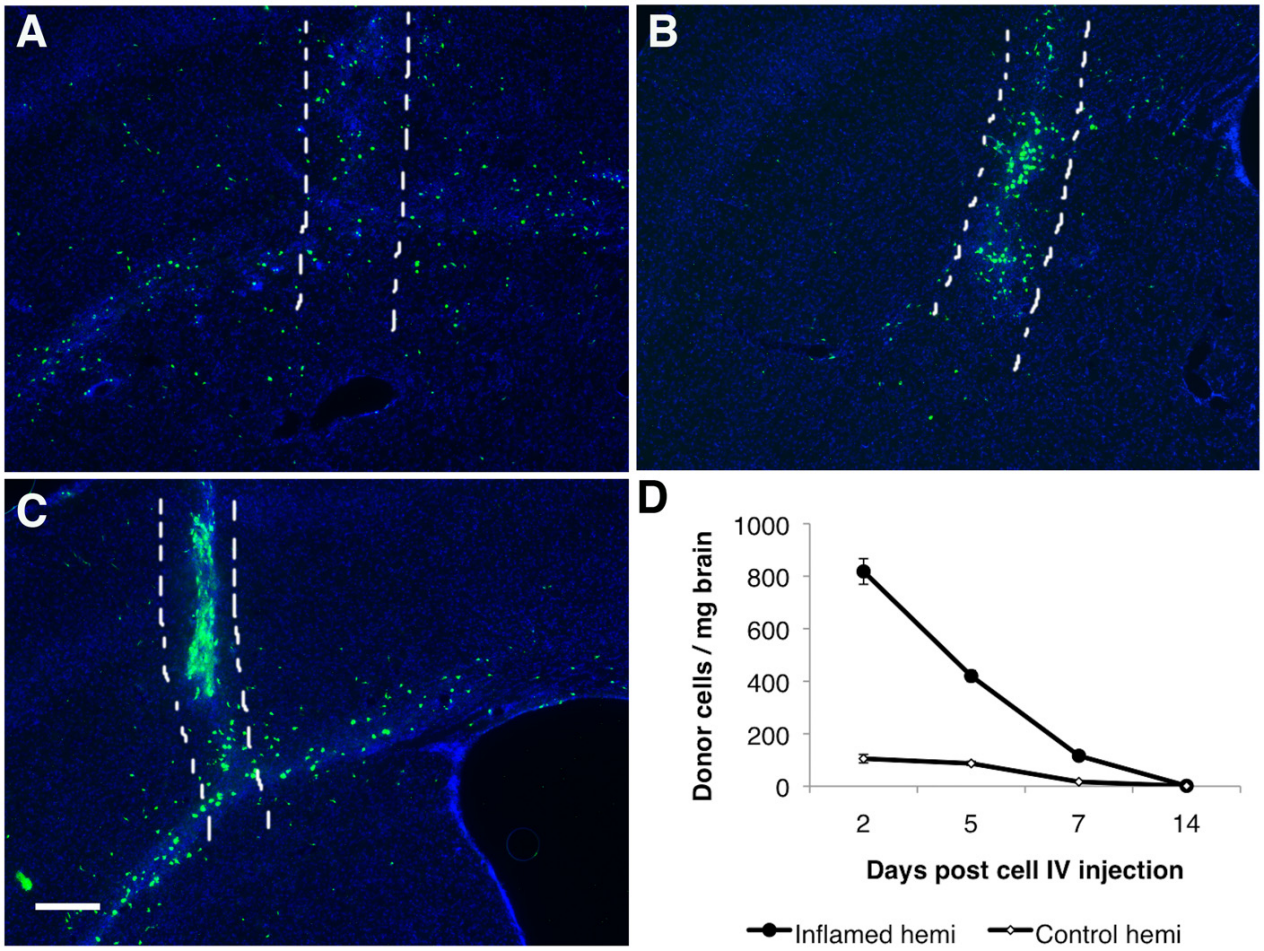
IV transferred monocytes ingression in brain. (A-C) Representative sections show the distribution of GFP positive donor-derived monocytes in the LPS injected brain at day 2(A), day 5(B), and day 7(C) post monocytes IV transfer. The area between the two white dash lines indicated the physical needle insertion site. For panel A-C, bar represent 200 μm, original magnification ×40. (D) Number of donor cells detected in the LPS injected hemisphere (ipsi hemi = ipsilateral hemi) and in the other hemisphere (contral hemi = contralateral) decreased over time post monocyte IV transfer.

Histological results revealed that distributions of recruited monocytes in the brain altered at different time points following the induction of neuroinflammation. At 2 days post cell IV infusion, a large amount of recruited GFP-positive cells were detected in the injected hemisphere, in a broad and widely-distributed fashion (Fig 2A). There were also some GFP-positive cells detected in the contralateral (un-injected) hemisphere, mostly within the motor cortex at regions close to the injected hemisphere (data not shown). This distribution pattern strongly suggested that recruited monocytes were attracted to the CNS sites with inflammatory responses in a controlled fashioned, and weren’t just passively leaked in from the physical trauma on the BBB created by the microinjection needle, as the CNS responses would be confined to only the needle injection tract in such case [40]. By day 5, most GFP-positive cells appeared to be in close association to the needle insertion site (physical trauma site) (Fig 2B). By day 7, majority of the transmigrated cells were found along the needle tract (at a lower amount as compared to day 5), and within the corpus callosum of the LPS-injected hemisphere (Fig 2C). Quantification results showed that recruited donor cells in brain decreased over time as the animals recovered from LPS-induced acute neuroinflammation (Fig 2D).

**Fig 3:**
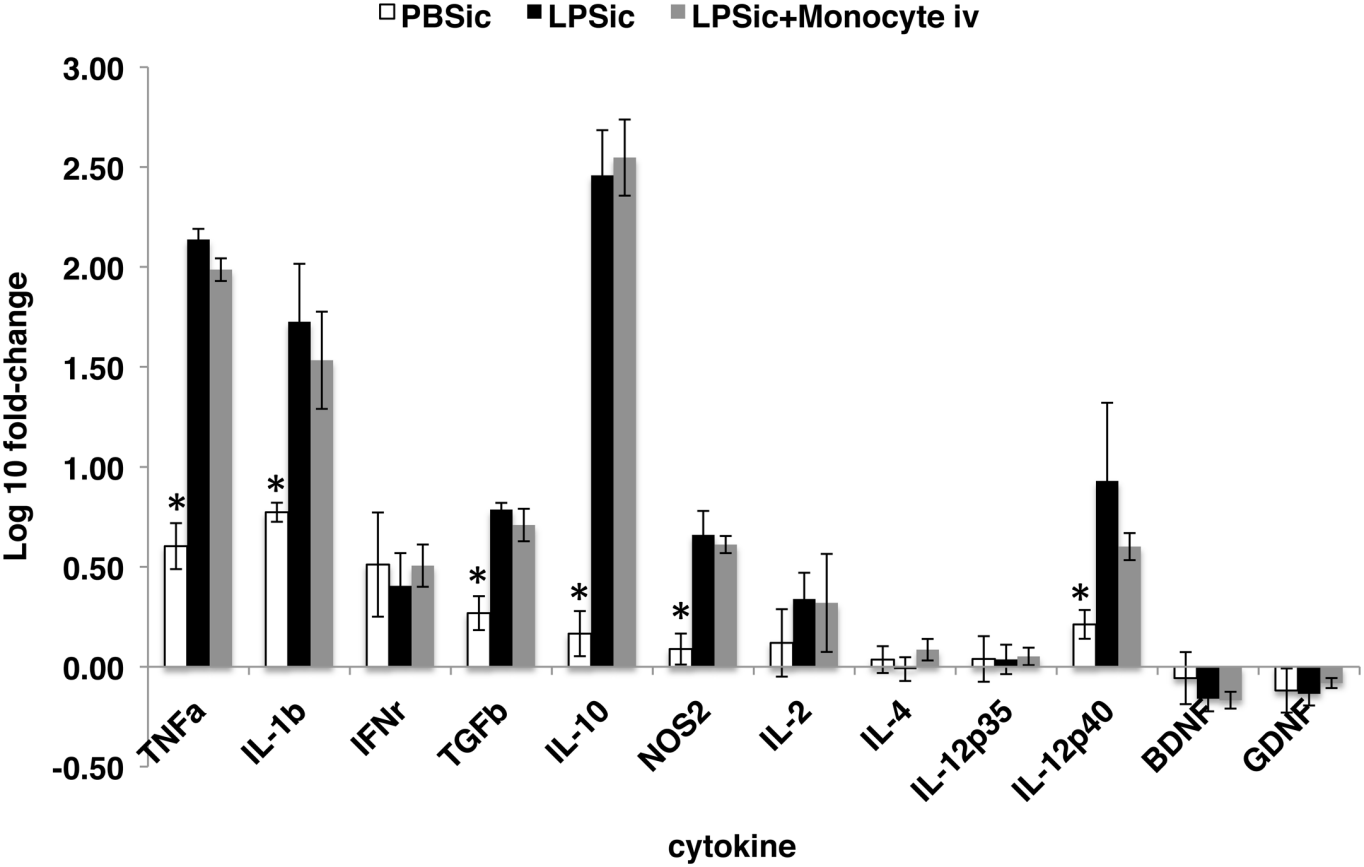
Expression analysis of selected cytokine genes in inflamed brain region. Transcription of TNFα, IL-1β, TGFβ, IL-10, IL-12p40 and NOS2 were significantly different in group PBSic compared to group LPSic and group LPSic+Monocytes iv (p-value < 0.05), but no difference was observed between group LPSic and group LPSic+Monocyte iv, indicating the presence of additional IV infused monocytes in circulation did not aggravate neuroinflammation. PBSic = sham control in which animals received PBS ICI. LPSic = inflammation control in which animals received LPS ICI. LPSic + Monocyte iv = animals received LPS ICI 24 hours prior to monocytes IV transfer. Cytokine gene transcription levels were measured at 72 hours post ICI. * = p-value < 0.05, unpaired *t* test. Final data presented here represents mean values +/- SD.

The amount of monocytes recruited to the damaged CNS tissues is tightly correlated to the degree of inflammation [14, 40]. Nevertheless, infiltrating monocytes has been related to several CNS disease progressions, including multiple sclerosis and HIV-associated neurocognitive disorders [14, 16]. Whether or not the presence of additional monocytes introduced by adoptive transfer to circulation would aggravate neuroinflammation was unclear. Therefore, quantitative RT-PCR analysis was conducted at 72 hrs following ICI delivery of LPS (48 hrs post cell IV infusion). As compared to control animals that received ICI delivery of PBS, significant increase of gene transcription was detected in the brains of animals received LPS, including tumor necrosis factor alpha (TNF-α), interleukin 1 beta (IL-1β), transforming growth factor beta (TGFβ), interleukin 10 (IL-10), nitric oxide synthase 2 (NOS_2_), and interleukin 12 subunit beta (IL-12p40) (Fig 3). However, no significant difference in cytokine gene transcription was observed between animals that received no cells and those received exogenous monocytes IV transfers at 24 hrs post ICI delivery of LPS (Fig 3), suggesting the transient presence of additional IV-transferred monocytes in circulation did not subsequently aggravate LPS-induced neuroinflammation.

### Enhancement of Monocyte Entry into Inflamed Brain Tissue

**Fig 4:**
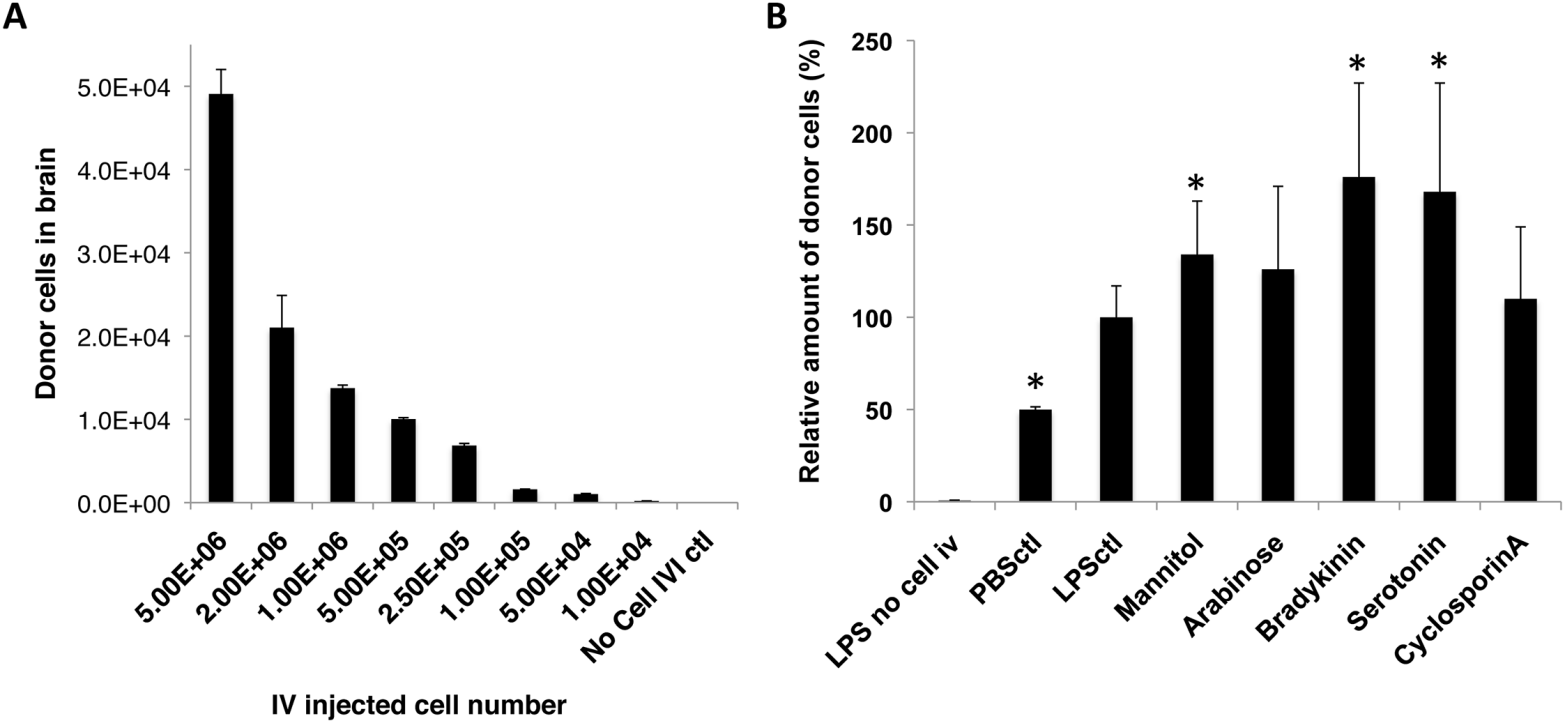
Enhanced monocytes entry into the inflamed brain. The amount of IV infused monocytes recruited to the inflamed brain regions could be enhanced by (A) increasing IV transferred monocyte amounts, and (B) transiently disrupting BBB by chemical agents. (A) The number of recruited donor-derived monocytes in the brain was positively correlated to the number of IV infused cells, as analyzed by Pearson correlation coefficient, with R = 0.9932, and R^2^ = 0.9864. No Cell IVI control = Control animal that received LPS ICI but no monocyte IV. (B) Mannitol, Bradykinin, and Serotonin enhanced entry of IV transferred monocytes into LPS-inflamed brain tissue by 134%± 29%, 176%± 51%, and 168± 59% compared to the group that received no BBBD reagents (LPSctl, p value < 0.05, unpaired *t* test). Two additional control groups were included: LPS no cell i.v. = mice that received LPS ICI but no cells, and PBSctl = mice that received PBS ICI, followed by monocyte IV transfer. Data was analyzed by unpaired *t* test (to LPS no cell i.v. control) and one-way ANOVA (among all test groups), with resulting p-value <0.05 (*) deemed as significant. No difference was found in groups treated with Arabinose and Cyclosporin A (p value > 0.05, unpaired *t* test). Final data presented here represents mean values ± SD.

One limitation of conducting cell adoptive transfer without preconditioning by lethal irradiation is that upon their entry into the circulation, these cells are diluted with endogenous cells; and as stated above, the amount of immune cells recruited to the target sites is fixed by the degree of inflammation. Therefore, we hypothesized that by rising exogenous to endogenous monocyte ratio, more transferred monocytes might be able to reach the affected brain regions. To test this, different numbers of monocytes ranged from 1×10^4^ to 5×10^6^ were infused into recipient mice that had received a fixed dose of LPS ICI (5μg) 24 hrs earlier. As the result, the number of donor-derived cells reaching the inflamed brain region was found to be positively correlated with the initial number of cells injected IV (R = 0.9922, R^2^ = 0.9845; Pearson Correlation Coefficient) (Fig 4A). Hence by increasing the number of initial IV transferred cells, it is possible to bring up vehicle concentrations at the target site for more effective treatments.

The permeability of the BBB can be transiently increased through the use of chemical agents to disrupt the barrier [22, 29–32]. Our previous study demonstrated enhanced entry of blood circulating monocytes (infused via common carotid artery, CCA) into the brain in steady physiological state, following transient disruption of the BBB using Mannitol and Bradykinin [22], suggesting the possibility of increasing brain entry of the cell vehicles by transiently modulating BBB permeability. To further optimize BBBD agent-facilitated enhancement of IV-infused cell vehicles entry into the CNS under inflammatory conditions, Bradykinin, Mannitol, and several other BBBD agents that could temporarily increase the permeability of the BBB via different mechanisms were tested and compared. As shown in Fig 4B, Mannitol, Bradykinin and Serotonin all showed statistically significant enhancement in the amount of recruited donor cells in the inflamed brain region at 48 hrs following cell IV infusion (p<0.05). Interestingly, the two calcium-channel modulating agents, Bradykinin and Serotonin, showed the highest enhancement in facilitating monocytes transmigration into the inflamed brains (176% ± 51% and 168% ± 59%), followed by the osmotic agent Mannitol (134% ± 29%) (Fig 4B).

### Recruited Monocyte Differentiated into IBA-1 Positive Cells with Microglia Morphology in the Brain

**Fig 5:**
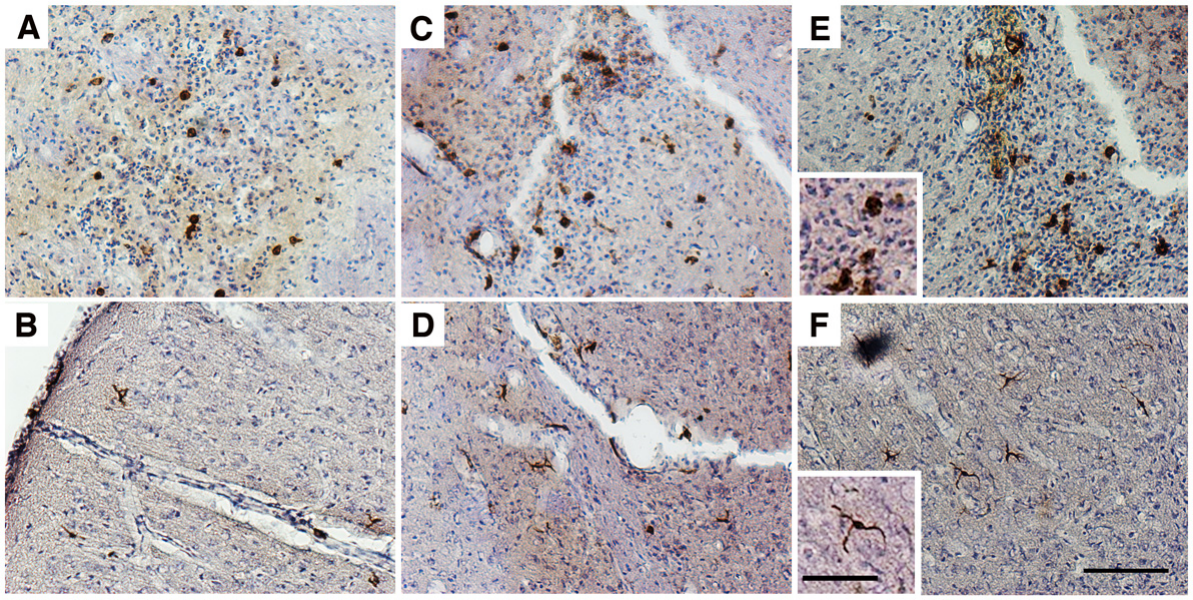
Recruited monocytes matured into cells with two distinguish morphologies in the brain. Two types of GFP positive donor-derived monocytes with very distinct morphologies were identified in the inflamed brain at day 2 (A& B), day 5 (C& D), and day 7 (E& F) post monocytes IVI. Panels A, C, and E show cells with big and round morphology that were often detected along the LPS injection tract, while panels B, D, and F show microglia-like, highly branched cells that were usually found in the cortical regions, further away from the needle tract. GFP positive cells were visualized by IHC staining with a GFP-specific primary antibody and a biotin-conjugated secondary antibody; Bars represent 100 μm (A-F) and 25 μm (insets). Original magnification ×100 for panel A-F.

**Fig 6:**
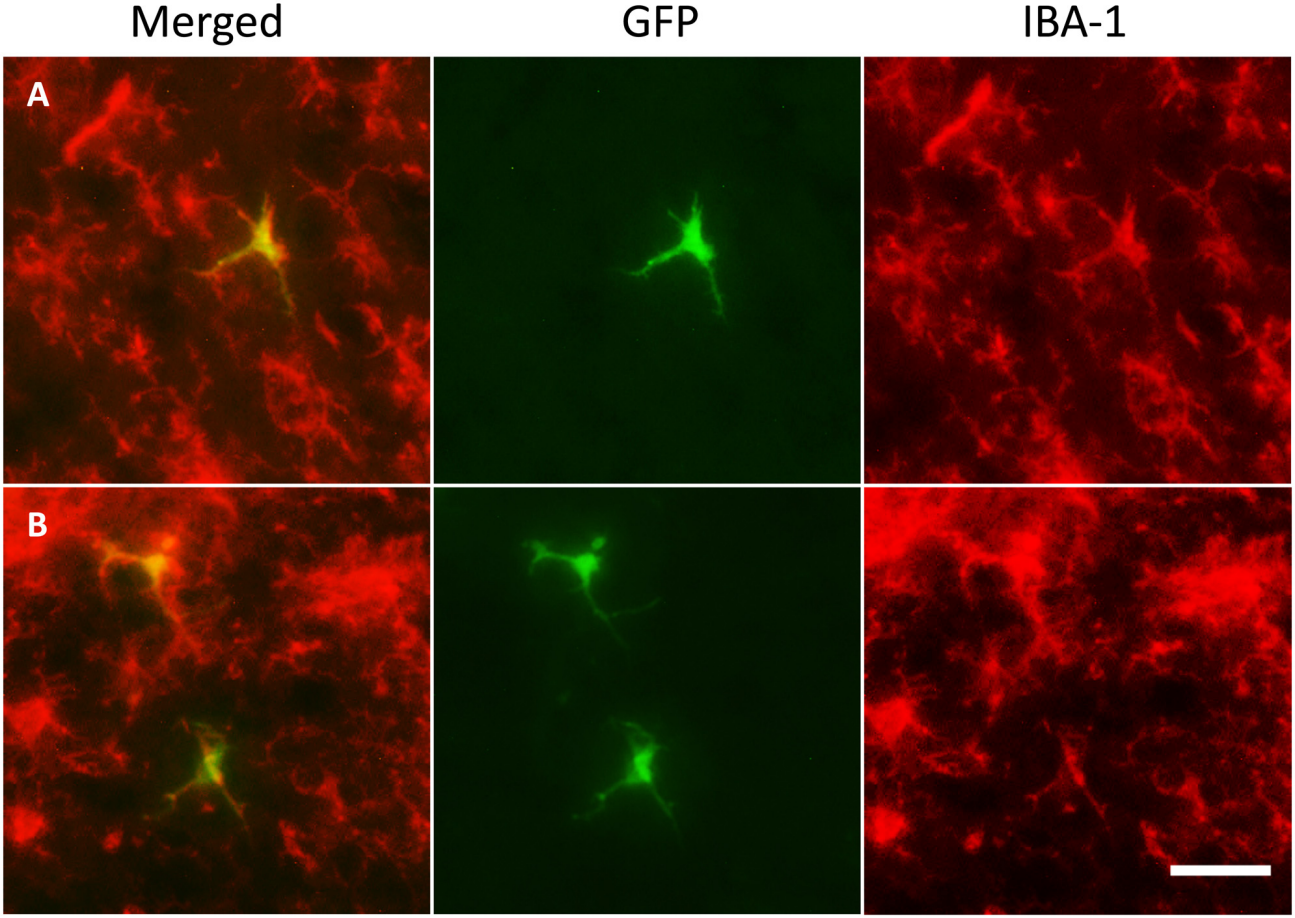
Recruited monocytes differentiated into IBA-1 positive cells with microglia morphology in the brain. GFP positive donor-derived cells (green) with a highly branched, microglia-like morphology located throughout the cortical region were Iba-1 positive (red) by day 5 (A) and day 7 (B) following IV adoptive transfer of monocytes. Bar represent 25 μm for all panel. Original magnification ×200 for panel A-F.

Histological analysis revealed that at 48 hours post cell infusion, GFP-positive cells recruited to the inflamed brain had a relatively homogeneous morphology, with majority of them being round (Fig 5A), and a few just slightly branched (Fig 5B). By day 5, most of donor-derived cells around the needle tract had a large and round appearance (Fig 5C), whereas cells in the cortex region started to show a more elongated and branched morphology (Fig 5D). By day 7, large and round cells were still detected around the needle tract (Fig 5E), and a small number of highly branched donor cells were also detected, mostly in the cortex regions (Fig 5F). Immunofluorescent analysis further revealed that a small portion of the donor cells had become Iba-1 positive by day 5 and day 7 post cell injection (Fig 6A and 6B), with all of them showing branched morphology, and were detected mostly in the cortex region further away from the microinjection site. Differentiation of recruited donor cells into GFAP positive astrocytes in brain was not detected under the described conditions (S2 Fig). qPCR results indicated the presence of a low amount of recruited donor cells in the LPS injected hemisphere at day 14 (Fig 2D), whereas no donor cell was detectable in the peripheral tissues including liver, lungs and spleen (data not show), suggesting that a small population of recruited donor cells were able to last in the brain after animals were recovered from acute neuroinflammation.

### Monocyte-Mediated Delivery of Nanoparticle SHP30 and Exogenous GFP Gene into the Inflamed Brain Tissues

Finally, we set out to determine the ability of “cargo” carriage monocytes to enter the brain. Two types of “cargos” were tested in this part of the study: SHP30 and GFP gene. SHP30 is a SPIO nanoparticle (SPION) that is 30nm in diameter. It was used to test the ability of monocytes to carry and transport nano-scaled agents (e.g. nano-formulated medicines or drug carriers) into the brain. On the other hand, GFP reporter gene was transferred into monocyte by an HIV-1-based vector D101, which allows gene integration into cell chromosome for permanent expression.

**Fig 7:**
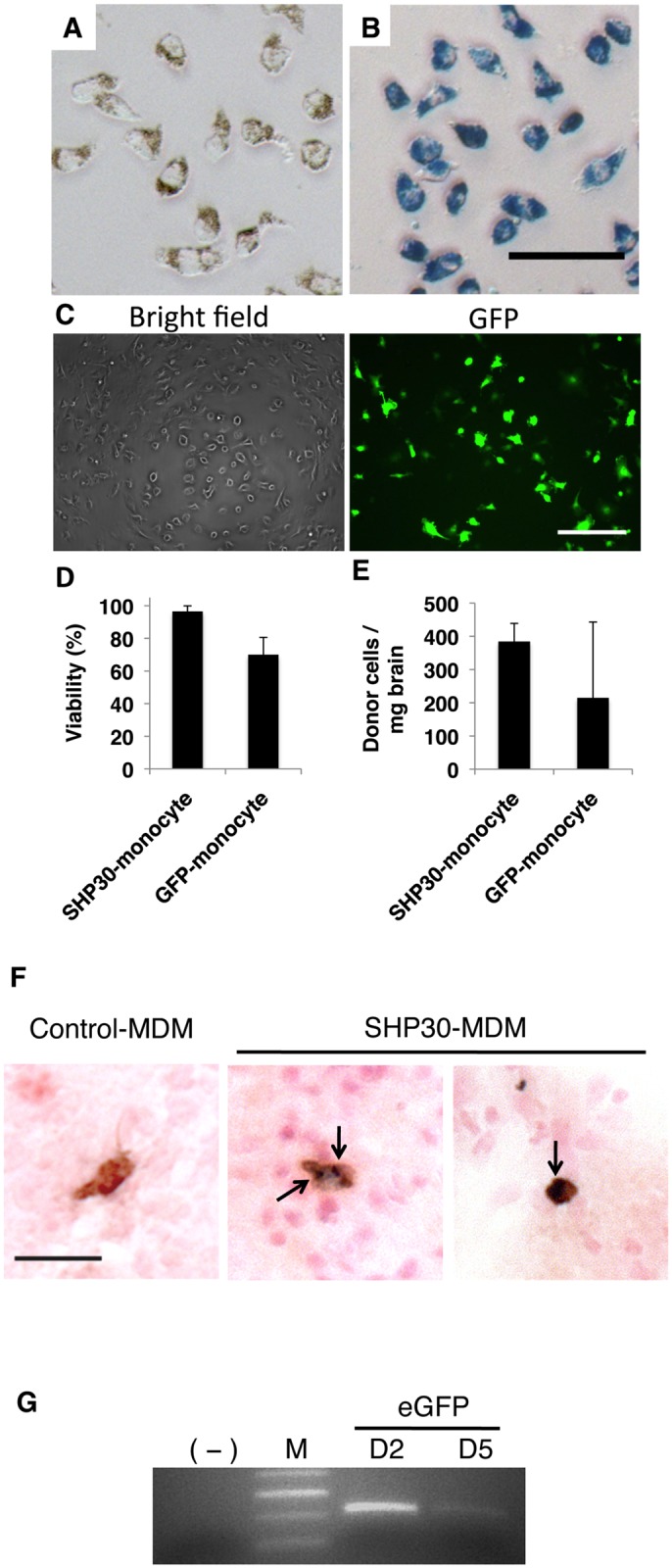
Monocyte-mediated delivery of SHP30 and GFP gene to the brain. (A&B) Overnight incubation of SHP30 with monocytes resulted in 100% SHP30 uptake efficiency, as observed by (A) the presence of brown SHP30 accumulates under the light microscopic fields, and by (B) the presence of Prussian blue pigments at monocytes cytoplasm following Prussian blue staining. (C) D101 LV-mediated GFP gene transfer resulted in around 36% transduction efficiency. (D) Viability of *ex vivo* modified monocytes following overnight incubation with 25 μg/mL SHP30 (SHP30-monocyte), and 90-minute transduction with D101 LV (GFP-monocyte). (E) SHP30-laden monocytes (SHP30-monocyte) or monocytes underwent D101 LV transduction (GFP-monocyte) were detected in the inflamed brain regions at 48 hours following cell IV infusion. (F) The presence of SHP30 (Dark blue, Prussian blue positive) was detected in the cytoplasm of the recruited donor-derived cells (Brown, GFP positive) in the brain. The arrow showed the presence of SHP30. (G) The presence of the exogenous GFP gene was detected in the inflamed brain by PCR at day 2 and day 5 following cell IV transfer. For Panel A &B, bar represents 50 μm, at original maginications x200. For panel C, bar represents 200 μm, at original magnification ×200. For panel F, bar represents 25 μm, at original magnification x400.

Overnight incubation of monocytes in SHP30 containing media resulted in 100% particle uptake efficiency, as determined by the presence of the particle accumulates (brown granules) (Fig 7A), and by Prussian blue positive pigments present in cell cytoplasm following Prussian blue staining (Fig 7B). LV-mediated GFP gene transfer, on the other hand, resulted in 36% of GFP-expressing monocytes (GFP-positive), as evaluated by fluorescent microscopy at 7 days post transduction (Fig 7C). After overnight SHP30 uptake, up to 96% SHP30-monocytes remained viable, as determined by Trypan blue exclusion assay (Fig 7D). Conversely, the 90 minutes LV transduction procedure resulted in nearly 30% cell death, with only 70% of transduced monocytes remained viable (Fig 7D). Immediately following the described *ex vivo* modifications, these male-donor derived monocytes were injected IV to female recipient animals bearing acute neuroinflammation induced by LPS IC the day before cell transfer. 48 hours following cell IV transfer, around 400 donor cells/mg brain for SHP30-monocytes, and 200 cells/mg brain for D101 LV transduced monocytes were detected in the inflamed brain tissues (Fig 7E), as determined by real-time qPCR measuring the amount of male-derived cells in the brain. In addition, cytoplasmic SHP30 carriage remained detectable in donor-derived monocytes following trafficking into the brain, as confirmed by IHC and Prussian blue staining (Fig 7F). Furthermore, the presence of the exogenous GFP gene in the inflamed brain region was confirmed by PCR at days 2 and 5 following IV infusion of the transduced cells (Fig 7G).

## Discussion

This study was designed to fully characterize the IV adoptive transferred monocytes for their migration into the brain using an acute neuroinflammation mouse model, and thus to establish the optimized conditions to facilitate the cell-based therapeutic delivery system for the CNS. We investigated infused monocytes and MDM for their inflamed-brain homing efficiency, and established conditions that could enhance monocyte brain entry, followed by evaluating the transmigrated donor cell engraftment and differentiation in the brain, and finally, validated the ability of nano-scaled agents- and exogenous genes-carriage monocytes to enter the brain by testing with SHP30 and the GFP reporter gene.

To begin with, cells within the mononuclear phagocyte (MP) family vary largely in their degree of maturation and cellular functions, thus it is important to first identify the cell types that are more suitable as delivery vehicles, which should have the ability to accumulate in the brain target sites with high efficiency following adoptive transfer. Bone marrow is a preferred source of monocytes and MDM for many studies in mice, since it allows generating large quantities of mouse MDM through *in vitro* cultivation [3, 5, 7, 35]. However, it is not clear how the duration of *in vitro* cultivation affects MDM migratory efficiency *in vivo*. MDM have been reported to successfully deliver anti-viral drugs and therapeutic gene products into diseased CNS tissue after 10–14 days of *in vitro* cultivation in the presence of granulocyte macrophage colony stimulating factor (GM-CSF) or M-CSF [5, 7]. Freshly isolated bone marrow CD11b+ cells have also been used to deliver therapeutic gene products for Alzheimer’s disease in a mouse model [6]. A previous study of IV transferred monocytes in irradiated recipient animals concluded that the relative mature *ex vivo* manipulated monocytes migrated and engrafted with better efficiency in tissues [33]; whereas Xu *et al* reported that only freshly isolated CD11b+ monocytic cells could circulate freely and traffic efficiently to the inflamed retina, but not MDM cultured *in vitro* for 6 days [34]. Nevertheless, few studies have provided a clear, quantitative comparison of the transmigratory efficiency of these transferred cells into the inflamed brain. Therefore, this study was first conducted to determine the relationship between the duration of *in vitro* cultivation and the *in vivo* trafficking efficiency of cMDM to inflamed CNS tissue in recipient mice. Our results indicated that freshly isolated monocytes and cMDM with shorter cultivation times migrated into the inflamed brain with much higher efficiency compared to cells that had undergone prolonged cultivation in the presence of M-CSF. These findings suggested that the relatively immature MP population were more suitable to be used as cell vehicles targeting the brain through the non-invasive IV administration.

Infiltrating monocytes have been reported to play critical roles in several CNS disease progression involving inflammation and tissue damages [14, 16]. Therefore, safety evaluation on conducting monocyte-based therapy is essential. After adoptive transfer, IV infused cells entered the circulation and mix with endogenous monocytes. Under normal physiological condition, there is about one million monocytes in mice peripheral blood, and the number increases during inflammation and pathological conditions [41]. When exogenous monocytes were introduced, there were considerably more monocytes in circulation for a transient period. Quantitative assessment of the mRNA expression level of several pro- and anti-inflammatory cytokine in brain revealed that this transient increase of monocytes in circulation did not aggravate the degree of neuroinflammation.

One important aspect when developing a successful cell-based delivery system is to establish conditions that can increase the number of cell vehicles present at the target sites. Unlike BMT procedure in which the endogenous immune cells and their precursors are largely destroyed by lethal irradiation, monocytes introduced by IV adoptive transfer were diluted with the endogenous cell pool upon entering circulation. As reported, the number for recruited cells into the brain was tightly controlled by the degree of neuroinflammation [14, 40], therefore, we increased the amount of IV transferred monocytes to bring up the exogenous to endogenous cell ratio, and confirmed that by increasing the number of IV infused cells, more donor cells were recruited to the inflamed brain region. In addition, we have further established the conditions in this study to transiently disrupt the BBB using chemical agents Mannitol, Bradykinin, and Serotonin to increase transmigration of donor cells into inflamed brain tissue (1.3–1.8 fold increase on average, p < 0.05, unpaired student t-test). The underlying mechanisms of this BBBD reagent-facilitated enhancement is presently not clear, and additional tests as well as further condition optimizations are necessary in the future. Nevertheless, this data supported the notion to increase IV-infused cell vehicle concentration at target brain tissues by increasing initial amount of transferred cells, as well as transiently disrupting the BBB using chemical agents.

Recent parabiosis-studies suggested that blood circulating monocytes were not able to differentiate into brain resident microglia, as demonstrated in both the mouse facial axotomy and experimental autoimmune encephalitis models [12, 14]. However, another study applying LPS IC injection into the brain reported local microglia death within 6 hours around the injection site, followed by replenish of regional IBA-1 positive cells derived from infiltrating monocytes [39]. Based on our observation. IBA-1 positive donor-derived cells started to appear at day 2 following cell IV infusion, and more IBA-1 positive cells with microglia-like morphology were detected at later time points, with majority of them located at the cortex region. To which extent can these IBA-1 positive cells last beyond the test period (14 day) requires further investigations. Nevertheless, the ability of IV transferred monocytes to enter the brain and differentiate into functional CNS tissue cells was indeed validated in this study.

Finally, the ability of monocytes to carry “cargos” into the brain was tested. SHP30 and GFP gene were selected as representative nano-scaled agent and exogenous gene, respectively. The numbers of the cargo-laden monocytes detected in the brains were in fact lower than monocytes without *ex vivo* modification. This decreased in homing efficiency was somewhat expected. The “loading” system we applied required overnight incubation of monocyte culture with the nanoparticles SHP30, and LV-mediated gene transfer involved a 90-minute transduction procedure using centrifugation. As observed in the study, elongated *in vitro* cultivation time has an adverse effect on the homing efficiency of cMDM, hence the overnight incubation with SHP30 was expected to decrease the brain migration level of the carrier monocytes. On the other hand, LV-mediated gene transfer using the spin-infection method involving centrifugation have caused certain degree of physical damages to the cells, which subsequently decreased carrier cells’ *in vivo* brain-migration efficiency. It should be noticed that we have confirmed the presence of SHP30-carriage monocytes in the brain by using histological method. However, it has been very challenging when the same approach was applied to validate the physical presence of GFP gene-carriage monocytes in the brain. As observed *in vitro*, the GFP signal expressed by carrier monocytes at 48 hours post transduction were very weak. Together with only 36% transduction efficiency and the fact of limited number of transduced-cells to enter the brain, detection of GFP-monocytes by IHC method was difficult. Nevertheless, current finding from PCR analysis has clearly demonstrated the presence of the GFP gene in the brain. These results together emphasized the importance of further method optimizations on the “loading” of therapeutic agents, especially therapeutic genes, into monocytes, and warrant for more in-depth study to confirm the viability and biological functionality of these recruited carrier cells in target tissues in the future.

*In total*, we have established an optimized monocyte-based delivery system, and validated the ability of IV adoptively transferred SHP30-carriage monocytes to enter the brain. The findings from this study support the notion of using monocyte-based delivery system as an alternative therapeutic approach towards neurological disorders, and argue for more in-depth tests and developments of such systems in the future.

## Supporting Information

S1 Fig Condition optimization for exogenous MDM delivery into acutely inflamed brain tissue. (A) The number of donor-derived MDM detected in the inflamed brain region at 24 hours following cell IV injection. Cells were introduced into the recipient animals at indicated time points post LPS-induced acute neuroinflammation. (B) The persistence of the recruited IV-infused donor cells in the inflamed brain of the recipient animals. Each experiment was independently performed 3 times, with 1 animal/group/experiment. Final data presented mean values ± SD. Results wer analyzed by one-way ANOVA, with resulting p-value <0.05.
(TIF)

S2 Fig Distribution of IV infused monocytes and brain resident astrocytes in the inflamed brain. Distribution of recruited IV-infused GFP positive donor monocytes (Green) and GFAP positive brain resident astrocytes (Red). (A&B) Brain tissue was analyzed at day 5 (A) and day 7 (B) post monocyte IV transfer. Recruited donor-monocytes were found to be in close association to the site in which astrocyte reactivity developed the most along the needle injection tract. (C) No GFAP expression was observed on the recruited IV-infused monocytes, indicating that recruited donor-monocytes do not differentiate into astrocytes after entering the inflamed brain. For panel A and B, scale bar = 100 μm, original magnification x100. For panel C, scale bar = 50μm, original magnification x200.
(TIF)

S1 Text Condition optimization for exogenous MDM delivery into acutely inflamed brain tissues. Initial experiments were conducted with MDM cultured for 5 days with M-CSF; this is reflective of prior works suggesting that the ratio of monocytes to mature macrophage reached its’ highest at that time point [35]. Five million D5-cMDM from male donor C57BL6 mice were injected IV at 6, 18, 24, 48 and 72h, as well as 5 and 7 days after IC injection of LPS into female recipient C57BL6 mice. Brain tissues were first collected at 24 hours after cell injection. Quantification analysis was performed with real-time qPCR to determine the amount of male donor-derived cell genomic DNA (gDNA) in each test group. The amount of gDNA within the brain peaked when cells were introduced at 24 hours following LPS treatment (S1A Fig). We then examined the persistence of the donor-derived MDM after the cells entered the inflamed brain. Brain tissues were collected at 1, 2, 3 and 7 days following MDM transfers into LPS ICI treated mice. The number of recruited donor-derived cells peaked at 48h and decreased afterwards (S1B Fig).
(DOCX)
