# Supplementary figures and images for: Monocyte Trafficking, Engraftment, and Delivery of Nanoparticles and an Exogenous Gene into the Acutely Inflamed Brain Tissue – Evaluations on Monocyte-Based Delivery System for the Central Nervous System
Source: PLoS One. 2016 Apr 26;11(4):e0154022. doi: 10.1371/journal.pone.0154022 (PMC4846033; doi:10.1371/journal.pone.0154022)

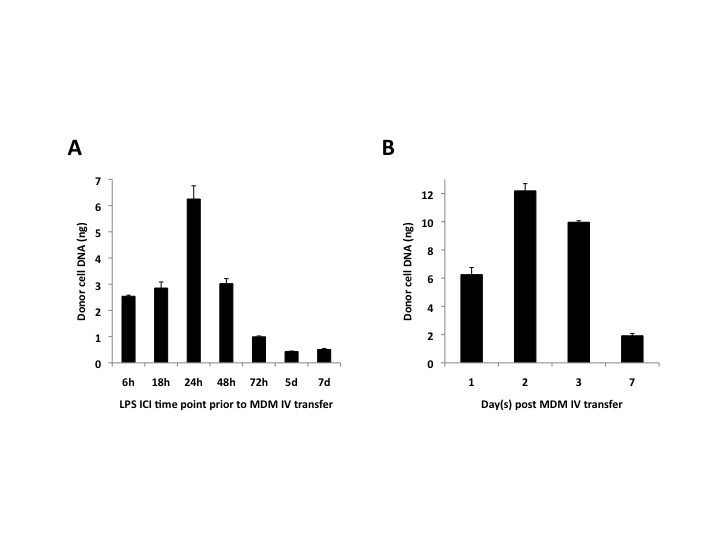

Supplement: S1 Fig — (A) The number of donor-derived MDM detected in the inflamed brain region at 24 hours following cell IV injection. Cells were introduced into the recipient animals at indicated time points post LPS-induced acute neuroinflammation. (B) The persistence of the recruited IV-infused donor cells in the inflamed brain of the recipient animals. Each experiment was independently performed 3 times, with 1 animal/group/experiment. Final data presented mean values ± SD. Results wer analyzed by one-way ANOVA, with resulting p-value <0.05. (TIF) [file pone.0154022.s001.tif]

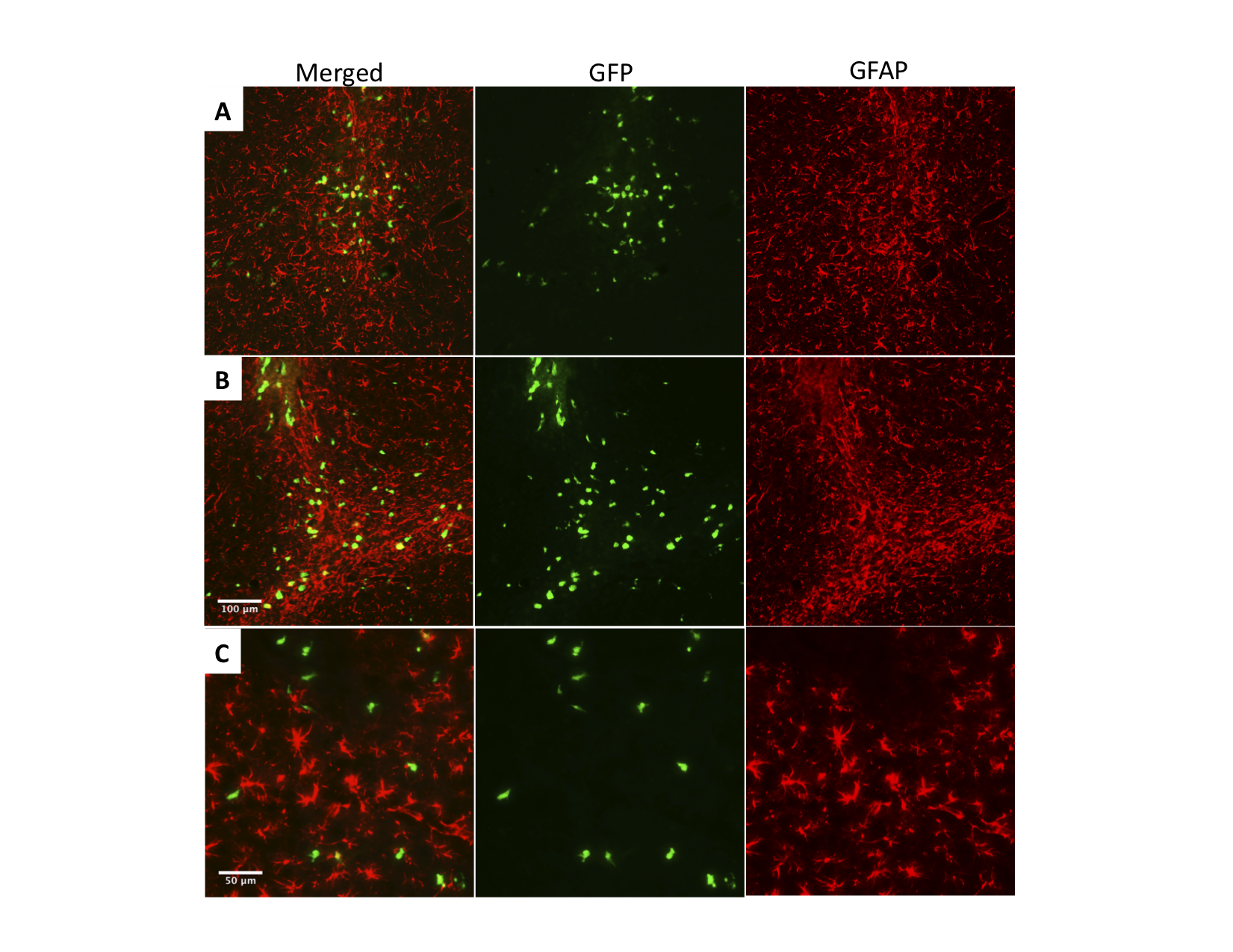

Supplement: S2 Fig — Distribution of recruited IV-infused GFP positive donor monocytes (Green) and GFAP positive brain resident astrocytes (Red). (A&B) Brain tissue was analyzed at day 5 (A) and day 7 (B) post monocyte IV transfer. Recruited donor-monocytes were found to be in close association to the site in which astrocyte reactivity developed the most along the needle injection tract. (C) No GFAP expression was observed on the recruited IV-infused monocytes, indicating that recruited donor-monocytes do not differentiate into astrocytes after entering the inflamed brain. For panel A and B, scale bar = 100 μm, original magnification x100. For panel C, scale bar = 50μm, original magnification x200. (TIF) [file pone.0154022.s002.tif]
