# Supplementary material for: Monocyte Trafficking, Engraftment, and Delivery of Nanoparticles and an Exogenous Gene into the Acutely Inflamed Brain Tissue – Evaluations on Monocyte-Based Delivery System for the Central Nervous System
Source: PLoS One. 2016 Apr 26;11(4):e0154022. doi: 10.1371/journal.pone.0154022 (PMC4846033; doi:10.1371/journal.pone.0154022)
Supplement: S1 Text — Initial experiments were conducted with MDM cultured for 5 days with M-CSF; this is reflective of prior works suggesting that the ratio of monocytes to mature macrophage reached its’ highest at that time point [35]. Five million D5-cMDM from male donor C57BL6 mice were injected IV at 6, 18, 24, 48 and 72h, as well as 5 and 7 days after IC injection of LPS into female recipient C57BL6 mice. Brain tissues were first collected at 24 hours after cell injection. Quantification analysis was performed with real-time qPCR to determine the amount of male donor-derived cell genomic DNA (gDNA) in each test group. The amount of gDNA within the brain peaked when cells were introduced at 24 hours following LPS treatment (S1A Fig). We then examined the persistence of the donor-derived MDM after the cells entered the inflamed brain. Brain tissues were collected at 1, 2, 3 and 7 days following MDM transfers into LPS ICI treated mice. The number of recruited donor-derived cells peaked at 48h and decreased afterwards (S1B Fig). (DOCX) [file pone.0154022.s003.docx]

**S1 Text**

Initial experiments were conducted with MDM cultured for 5 days with M-CSF; this is reflective of prior works suggesting that the ratio of monocytes to mature macrophage reached its’ highest at that time point([1](#_ENREF_1)). Five million D5-cMDM from male donor C57BL6 mice were injected IV at 6, 18, 24, 48 and 72h, as well as 5 and 7 days after IC injection of LPS into female recipient C57BL6 mice. Brain tissues were first collected at 24 hours after cell injection. Quantification analysis was performed with real-time qPCR to determine the amount of male donor-derived cell genomic DNA (gDNA) in each test group. The amount of gDNA within the brain peaked when cells were introduced at 24 hours following LPS treatment **(S1 Figure A)**.

We then examined the persistence of the donor-derived MDM after the cells entered the inflamed brain. Brain tissues were collected at 1, 2, 3 and 7 days following MDM transfers into LPS ICI treated mice. The number of recruited donor-derived cells peaked at 48h and decreased afterwards **(S1 Figure B)**.

**S1 Figure. Condition optimization for exogenous MDM delivery into acutely inflamed brain tissue.** (A) The number of donor-derived MDM detected in the inflamed brain region at 24 hours following cell IV injection. Cells were introduced into the recipient animals at indicated time points post LPS-induced acute neuroinflammation. (B) The persistence of the recruited IV-infused donor cells in the inflamed brain of the recipient animals. Each experiment was independently performed 3 times, with 1 animal/group/experiment. Final data presented mean values ± SD. Results wer analyzed by one-way ANOVA, with resulting p-value <0.05.

**S2 Text**

**S2 Figure. Distribution of IV-infused monocytes and brain resident astrocytes in the inflamed brain.**

Distribution of recruited IV-infused GFP positive donor monocytes (Green) and GFAP positive brain resident astrocytes (Red). (A&B) Brain tissue was analyzed at day 5 (A) and day 7 (B) post monocyte IV transfer. Recruited donor-monocytes were found to be in close association to the site in which astrocyte reactivity developed the most along the needle injection tract. (C) No GFAP expression was observed on the recruited IV-infused monocytes, indicating that recruited donor-monocytes do not differentiate into astrocytes after entering the inflamed brain. For panel A and B, scale bar = 100 μm, original magnification x100. For panel C, scale bar = 50μm, original magnification x200.

**Reference:**

1. Francke A, Herold J, Weinert S, Strasser RH, Braun-Dullaeus RC. Generation of mature murine monocytes from heterogeneous bone marrow and description of their properties. J Histochem Cytochem. [Research Support, Non-U.S. Gov't]. 2011 Sep;59(9):813-25.
